# Comparison of Stemness and Gene Expression between Gingiva and Dental Follicles in Children

**DOI:** 10.1155/2016/8596520

**Published:** 2016-08-30

**Authors:** Chung-Min Kang, Seong-Oh Kim, Mijeong Jeon, Hyung-Jun Choi, Han-Sung Jung, Jae-Ho Lee

**Affiliations:** ^1^Department of Pediatric Dentistry, College of Dentistry, Yonsei University, Seoul, Republic of Korea; ^2^Oral Science Research Center, College of Dentistry, Yonsei University, Seoul, Republic of Korea; ^3^Department of Oral Biology, Division of Histology, College of Dentistry, Yonsei University, Seoul, Republic of Korea

## Abstract

The aim of this study was to compare the differential gene expression and stemness in the human gingiva and dental follicles (DFs) according to their biological characteristics. Gingiva (*n* = 9) and DFs (*n* = 9) were collected from 18 children. Comparative gene expression profiles were collected using cDNA microarray. The expression of development, chemotaxis, mesenchymal stem cells (MSCs), and induced pluripotent stem cells (iPSs) related genes was assessed by quantitative reverse transcription-polymerase chain reaction (qRT-PCR). Histological analysis was performed using hematoxylin-eosin and immunohistochemical staining. Gingiva had greater expression of genes related to keratinization, ectodermal development, and chemotaxis whereas DFs exhibited higher expression levels of genes related to tooth and embryo development. qRT-PCR analysis showed that the expression levels of iPSc factors including* SOX2*,* KLF4*, and* C-MYC* were 58.5 ± 26.3, 12.4 ± 3.5, and 12.2 ± 1.9 times higher in gingiva and* VCAM1* (CD146) and* ALCAM* (CD166) were 33.5 ± 6.9 and 4.3 ± 0.8 times higher in DFs. Genes related to MSCs markers including* CD13*,* CD34*,* CD73*,* CD90*, and* CD105* were expressed at higher levels in DFs. The results of qRT-PCR and IHC staining supported the microarray analysis results. Interestingly, this study demonstrated transcription factors of iPS cells were expressed at higher levels in the gingiva. Given the minimal surgical discomfort and simple accessibility, gingiva is a good candidate stem cell source in regenerative dentistry.

## 1. Introduction

Tissue engineering using mesenchymal stem cells (MSCs) is one of the most promising therapeutic strategies because MSCs have a high proliferation potential and may be manipulated to permit differentiation before transplantation [1, 2]. To date, different human dental stem cells have been isolated from dental pulp stem cells (DPSCs) [3], stem cells from exfoliated deciduous teeth (SHED) [4], periodontal ligament (PDL) stem cells [5], stem cells from apical papilla (SCAP) [6], and dental follicle precursor cells (DFPCs) [7]. Recently, mounting evidence suggests that gingiva derived mesenchymal stem cells were isolated and characterized as having multilineage differentiation capacity and immunomodulatory properties [8]. The presence of stem cell populations in dental follicles and the gingiva was revealed recently, and the related gene expression patterns remain unclear.

The dental follicle (DF) tissue is a connective fibrous tissue sac surrounding the enamel organ and the dental papilla of the developing tooth germ [9]. The DF cells have been proposed to have the capacity to differentiate into periodontium consisting of cementum, alveolar bone, and PDL [10, 11]. Despite an ectomesenchymal origin similar to that of the DFs, the gingiva appears to exhibit distinct functional activities during the maintenance of tissue integrity and during inflammatory responses [12]. It possesses a unique scarless healing process after wounding instead of the scar formation that is frequently observed in damaged extraoral tissues. So gingival tissue is postulated to have distinctive characteristics that accelerate wound closure, suggesting unique stemness with the ability to induce directed differentiation and regeneration.

Although some efforts were made to identify the genes that are differentially expressed in the periodontium [12–14], the genetic differences between the gingiva and DFs remain unknown. Given the anatomical and functional differences between the two tissues, it is reasonable to assume that there are also differences in the gene expression patterns. Thus, genetic investigation related to epithelial-mesenchyme interaction between gingiva and dental follicle can provide critical importance in regulating cell population and signaling system in the regeneration of periodontium. The aim of this study is to compare the gene expression patterns of the gingiva and DFs to enhance our understanding of the distinct regenerative ability in gingiva and tissue differentiation capacity in DFs.

## 2. Materials and Methods

The Institutional Review Board of the Yonsei University Dental Hospital approved the experimental protocol (approval number 2-2015-0005). All the subjects or their guardians have provided written informed consent. We used procedures similar to that recently applied by Song et al. [15] and Lee et al. [14].

### 2.1. Tissue Sampling and RNA Isolation

Gingival tissues were collected from children (*n* = 9) (5 males and 4 females, aged 7–12 years) with a healthy gingiva who underwent surgical gingival resection for the extraction of a supernumerary tooth, for odontoma, or for orthodontic reasons. The DF tissues were obtained from children (*n* = 9) (6 males and 3 females, aged 6–8 years), and they were separated from the coronal portion of the tooth during the extraction of supernumerary teeth. In DF, a piece of gingival tissue around the extraction socket was carefully curetted. These samples were immediately frozen and stored in liquid nitrogen. We used fresh tissue instead of cultured cells because, at the tissue level, gene expression reflects simultaneous profiles of many genes and can provide additional insights into the physiological processes or tissue-specific functions that are mediated by the coordinated action of sets of genes. Gingiva and DFs were immediately submerged in RLT buffer, which is a component of the RNeasy Fibrous Mini kit® (Qiagen, CA, USA). Prior to the RNA extraction, the tissues in RLT buffer were homogenized using a Bullet Blender® Bead (Next Advanced, Inc., NY, USA). Total RNA was extracted from gingiva and DFs using the RNeasy Fibrous Mini kit (Qiagen, USA) according to the manufacturer's instructions. The extracted RNA was eluted in 25 *μ*L of sterile water. RNA concentrations were measured from absorbance values at a wavelength of 260 nm using a spectrophotometer (NanoDrop ND-2000, Thermo Scientific, IL, USA). The RNA samples used in this study had 260/280 ratios of at least 1.8.

### 2.2. cDNA Microarray Construction and Data Analysis

Global gene expression analyses were performed using Affymetrix Gene Chip® Human Gene 1.0 ST oligonucleotide arrays (Affymetrix Inc., CA, USA). The average amount of RNA isolated from the gingiva and DFs was 1 *μ*g. As recommended by the manufacturer's protocol, 300 ng of total RNA from each sample was converted to double-stranded cDNA. The cDNA was regenerated via random-primed reverse transcription using a dNTP mix containing dUTP. The fragmented, end-labeled cDNA was hybridized to the Gene Chip® Human Gene 1.0 ST array for 16 hours at 45°C and 60 rpm with a terminal transferase reaction incorporating a biotinylated dideoxynucleotide. After hybridization, the chips were stained and washed in a Genechip Fluidics Station 450® (Affymetrix) and scanned using a Genechip Array scanner 3000 G7® (Affymetrix). To determine whether genes were differentially expressed between the separated tissue groups, a one-way ANOVA was performed on the Robust Multi-Average (RMA) expression values. A multiple testing correction was applied to the *p* values of the *F*-statistics to adjust the false discovery rate. Genes with adjusted *F*-statistic *p* values <0.05 were extracted. Genes that were highly expressed in the gingiva or DFs and that exhibited differences greater than 4-fold between the signal value of the control and the test group were selected for further study. These genes were then classified based on the information related to gene function that is available in Gene Ontology from the KEGG Pathway database (http://david.abcc.ncifcrf.gov/home.jsp). This microarray data set was approved by the Gene Expression Omnibus (GEO) (http://www.ncbi.nlm.nih.gov/geo/); the GEO accession numbers of the data set are GSE58480 (gingiva) and GSE51342 (dental follicle).

### 2.3. Quantitative RT-PCR

The single-stranded cDNA required for the polymerase chain reaction (PCR) analysis was produced using 500 ng of extracted total RNA as a template for reverse transcription (RT) (Superscript III Reverse Transcriptase and random primer, Invitrogen, UK). The RT reaction was incubated at 65°C for 5 minutes, then 25°C (5 min), 50°C (1 hr), and 70°C (15 min) to inactivate the activity of the reverse transcriptase. The synthesized cDNA was diluted 1 : 10 in distilled water and used as a template for quantitative RT-PCR using the ABI7300 RT-PCR system (Applied Biosystems, Warrington, UK). The samples were prepared in triplicate with a volume of 25 *μ*L containing 1x Universal TaqMan Master Mix (4369016, Applied Biosystems), the PCR primers at 0.9 *μ*M, and the diluted cDNA. The amplification conditions were 50°C for 2 minutes and 95°C for 10 minutes, followed by 40 cycles of 95°C for 15 seconds and 60°C for 1 minute. The following TaqMan gene expression assay primers (Applied Biosystems) were used: KRT6A, CXCL10, CSTA, AMBN, ADAM12, CXCL12, cMYC, KLF4, SOX2, CD106 (VCAM1), CD166 (ALCAM), and 18S rRNA. We selected known genes that are representative of the two tissues and little-known genes that are involved in their physiological functions.

ABI 7300 SDS 1.3.1 software (Applied Biosystems) recorded the fluorescence intensity of the reporter and quencher dyes, and results are plotted versus time, represented by the cycle number. The amplification plots were examined during the early log phase of product accumulation above background (the threshold cycle number, Ct) to obtain a precise quantification of initial target. The Ct values (the threshold cycle (Ct) number) were subsequently used to determine ΔCt values (ΔCt = Ct of the gene minus Ct of the 18S rRNA control). Relative expressions were expressed as the relative change by applying the equation 2^−ΔΔCt^ (ΔΔCt; differences in ΔCt values). All these quantitative RT-PCR procedures were done obtaining triplicated data. The results were analyzed using SPSS 20 software (SPSS Inc., IL, USA). Statistical differences were calculated by Mann–Whitney *U* tests, and *p* < 0.05 was considered statistically significant. The specific primer assay ID and product sizes for each gene are listed in Table 1.

### 2.4. Immunohistochemical Staining

For immunohistochemical staining, gingival tissue and DF tissue were fixed in 10% buffered formalin for 1 day, embedded in paraffin, and then sectioned at a thickness of 3 *μ*m. The specimens were subjected to IHC staining with antibodies specific for CXCL10 (rabbit polyclonal, diluted 1 : 50; Ab9807, Abcam, Cambridge, UK), CSTA (rabbit polyclonal, diluted 1 : 2,000; Ab61223, Abcam), AMBN (rabbit polyclonal, diluted 1 : 200; Ab116347, Abcam), and CXCL12 (rabbit polyclonal, diluted 1 : 50; Ab9797, Abcam). Endogenous peroxidase activity was quenched via addition of 3% hydrogen peroxide. The sections were incubated in 5% bovine serum albumin to block nonspecific binding. The primary antibodies were diluted to facilitate optimal staining, and the sections were incubated overnight. After incubation, EnVision+ System HRP-Labeled Polymer anti-rabbit (K4003, Dako North America, Inc., CA, USA) was applied for 20 min. Color development was performed using labeled streptavidin biotin kits (Dako) according to the manufacturer's instructions.

## 3. Results

### 3.1. Gene Expression Profiles of the Gingiva and Dental Follicles

1,182 out of 33,297 (3.6%) genes exhibited an absolute expression change of at least 4-fold. The expression levels of 555 genes were 4-fold higher in the gingiva than in DFs, while the expression levels of 627 genes were at least 4-fold higher in DFs than in the gingiva. The overall data distribution and frequency were confirmed by density and box plots of the ratio of the standardized log intensity to the average intensity. Ultimately, 829 genes were analyzed further, with the exception of several genes with unknown biological functions. The data were further filtered, and the genes are listed in Tables 2 and 3 according to their relative biological functions. In the gingiva, the expression levels of 387 genes were upregulated by 4-fold or more in comparison to DFs, while the expression levels of 442 genes were upregulated by 4-fold in DFs in comparison to the gingiva.

### 3.2. Gene Ontology Analysis

**Figure 1:**
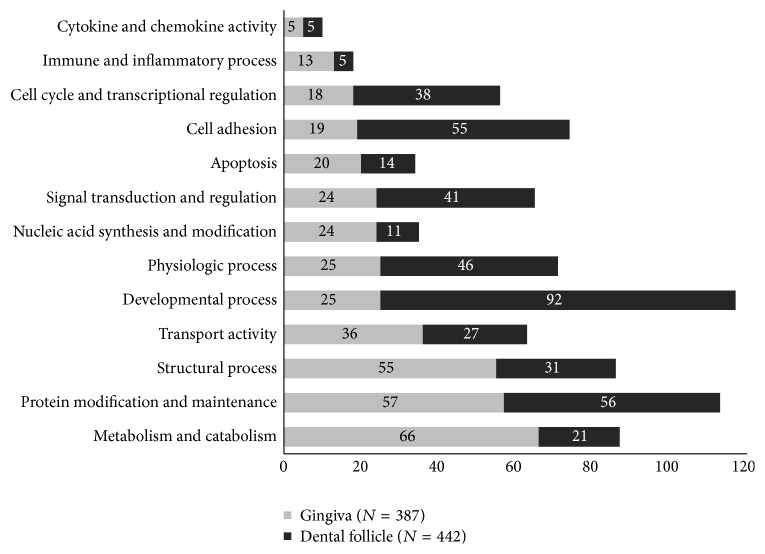
Main categories of genes expressed in the gingiva and dental follicles according to biological process. *x*-axis: the number of involved genes.

To identify the biological functions and features of the selected genes, the expression data sets were organized into Gene Ontology Consortium (GO) groups using the DAVID web-based tool. These genes were then classified based on information regarding gene function in gene ontology from the KEGG Pathway database.  Figure 1 shows GO classes for the two data sets analyzed (*F*-statistic *p* < 0.05).

A total of 66 genes encoding metabolic and catabolic process were expressed more abundantly in the gingiva than in the DFs. Fifty-five genes related to structural processes such as keratinization and cytoskeleton organization were expressed at higher levels in the gingiva. On the other hand, 92 developmental process-related genes were highly expressed in DFs as a result of biological processes including odontogenesis, ossification, and bone mineralization. Cell cycle-associated genes and signal transduction- and regulation-related genes were expressed at higher levels in DFs. These results are consistent with the occurrence of higher proliferation rates in DFs.

### 3.3. Confirmation of Gene Differential Expression Using Quantitative RT-PCR

**Figure 2:**
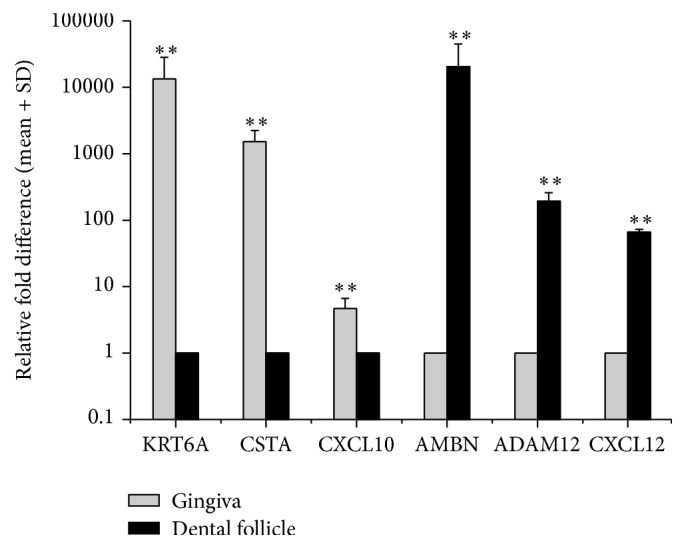
The relative difference in mRNA expression of six differentially expressed genes between the gingiva and dental follicles using quantitative RT-PCR. The data are presented as the mean + standard deviation and expressed as the relative change by applying the equation 2^−ΔΔCt^. *y*-axis: a log scale measure. ^*∗∗*^ *p* < 0.05.

Quantitative RT-PCR analysis verified the cDNA microarray results. Six genes for which the difference in expression levels between the gingiva and DFs was at least 4-fold were selected. Mann–Whitney “*U*” test was performed to correlate the relative change with differential expression as detected by PCR. The expression levels of* KRT6A*,* CSTA*, and* CXCL10* were 13406.7 ± 14962.8, 1524.4 ± 714.8, and 4.7 ± 2.0 times higher in gingiva, and* AMBN*,* ADAM12*, and* CXCL12* were 20585.4 ± 24267.0, 192.5 ± 66.5, and 66.0 ± 6.5 times higher in DFs (Figure 2). These results were consistent with the microarray results.

### 3.4. Verification of Array Results by Immunohistochemical Staining

**Figure 3:**
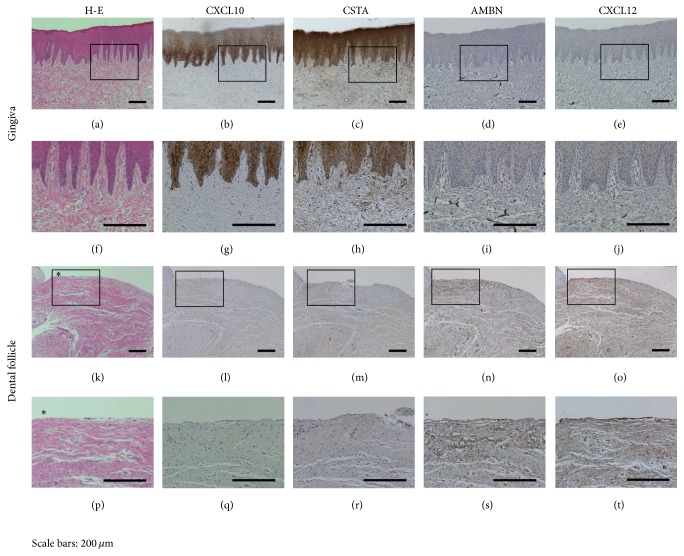
Verification of microarray results by immunohistochemical (IHC) staining. Hematoxylin-eosin staining in the gingiva (a, f) and dental follicles (DFs) (k, p) (asterisk: outer border neighboring alveolar bone). IHC staining for* CXCL10* in the gingiva (b, g) and DFs (l, q). IHC staining for* CSTA* in the gingiva (c, h) and DFs (m, r). The expression of* CXCL10* and* CSTA* was stained markedly in the gingival epithelium. The IHC staining for* AMBN* in the gingiva (d, i) and DFs (n, s). AMBN was stained around the outer layer of the DFs. The IHC staining for* CXCL12* in the gingiva (e, j) and dental follicles (o, t). CXCL12 was stained in both a cellular layer and the collagenous connective tissue of DFs (scale bars: 200 *μ*m).

The following four proteins were the targets of the IHC study:* CXCL10*,* CSTA*,* AMBN*, and* CXCL12* (Figure 3).* CXCL10* was broadly stained in the epithelial area of the gingiva.* CSTA* was strongly stained in all of the layers of the gingiva.* AMBN* was not stained in the gingiva but stained around the outer area of the DFs.* CXCL12* was stained in a single cellular layer and in the collagenous connective tissue of DFs. The results were consistent with those of the cDNA microarray analysis at the protein level.

### 3.5. Stemness Characterization by Surface Protein Markers

**Figure 4:**
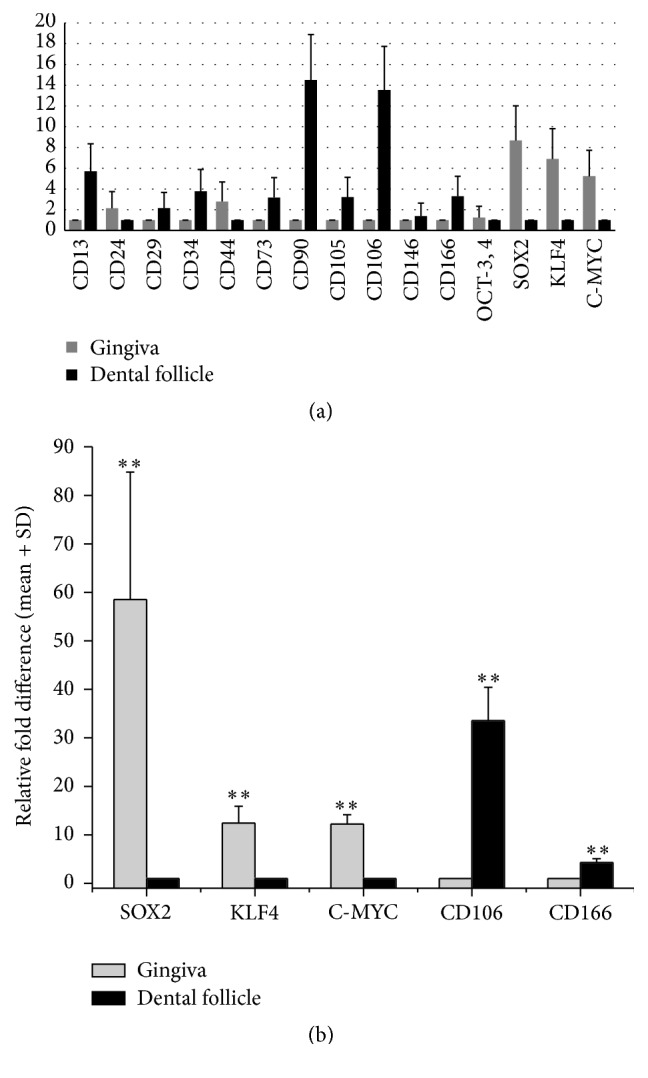
The relative gene expression of dental-derived stem cell and induced pluripotent stem cell markers using cDNA microarray (a). The relative fold difference in the expression of five stem cell marker genes between the gingiva and dental follicles using quantitative RT-PCR (b). The data are presented as the mean + standard deviation (a, b) and expressed as the relative change by applying the equation 2^−ΔΔCt^ (b). ^*∗∗*^ *p* < 0.05.

Based on previous studies, dental stem cells were characterized using surface protein markers [16, 17]. The comparative expression results for stem cell marker genes are listed in Figure 4(a). Our results indicated that DF tissue derived MSCs are a cell population that is more positive for mesenchymal MSC markers (including* CD13*,* CD34*,* CD73*,* CD90*, and* CD105*) according to the International Society for Cell Therapy [18]. The comparative expression of four induced pluripotent stem cells (iPSCs) marker genes (i.e.,* OCT-3*,* 4*,* SOX2*,* cMYC*, and* KLF4*) were expressed at higher levels in the gingiva. As a result of qRT-PCR,* SOX2*,* KLF4*, and* cMYC* appeared 58.5, 12.43, and 12.23 times higher from the gingiva and* VCAM1 (CD106)* and* ALCAM (CD166)* were 33.54 and 4.27 times higher in DFs (Figure 4(b)). However, OCT-3, 4 did not show a clear difference in comparison to the other markers (0.46-fold difference).

## 4. Discussion

In this study, a cDNA microarray comparison analysis was performed to focus on differences in the gene expression profiles of gingiva and DFs in children. The majority of genes (32,115 out of 33,297, 96.5%) showed similar expression level between the gingiva and DFs when using a 4-fold absolute change cutoff value. Most of those genes encoded cell adhesion proteins, proteins involved in structural processes, or proteins related to signal transduction and regulation. This finding suggests that the gingiva and DFs differentiate into different tissue later although they originate from an ectomesenchymal cell. This is likely due to the regulation of comparable intracellular signaling pathways. In contrast, approximately 4% of genes were differentially expressed above the selected threshold. While accounting for only a small portion of the whole gene array, these genes might contribute to the distinct biological functions and distinguish each other phenotypically and morphologically. To investigate this assumption, comparative gene expression was analyzed with respect to the biological functions of the genes.

In the gingiva,* KRT1*,* CSTA*, and* FLG* were expressed at significantly higher levels. The gingival epithelium is a stratified squamous keratinizing tissue, and these genes are related to keratinization or keratinocyte differentiation.* KRT1* marks the cornification pathway of differentiation and is expressed in keratinized areas [19].* CSTA* is one of the precursor proteins of the cornified cell envelope in keratinocytes and plays a role in epidermal development and maintenance [20].* FLG* is essential for the regulation of epidermal homeostasis and interacts with keratin intermediate filaments [21]. Epidermis and ectoderm development-related genes were strongly upregulated in the gingiva versus DFs.* KRT6B* and* KRT6A* were markedly upregulated in the gingiva, with 90.30- and 57.61-fold differential expression, respectively. These proteins are rapidly induced in wound-proximal epidermal keratinocytes after skin injury and regulate the migratory potential of skin keratinocytes during wound repair [22].* SCEL* may function in the assembly or regulation of proteins in the keratinized envelope [23]. The upregulation of these genes may indicate the existence of a fast turnover rate in the gingiva and may facilitate fibroblast proliferation, which is an important event for tissue repair.

The oral mucosa is affected by exposure to various extrinsic factors such as chemicals and microorganisms. Genes related to apoptosis and chemotaxis such as* CXCL10*,* CXCL17*,* ANLN*, and* CCL21* were strongly expressed in the gingiva.* CXCL10* is secreted by the keratinocytes and is a marker of the host immune response [24]. This chemokine plays an important role in the infiltration of Th1 cells and affects the gingiva by exacerbating periodontal disease [25]. The overexpression of these chemokines might be associated with the generation and delivery of immune and inflammatory responses in the gingiva.

On the other hand, genes related to tooth and embryo development exhibited significantly higher expression in DFs. These results are consistent with those of a previous DF gene expression study that compared DFs to the PDL [14]. The increased expression of* AMBN* indicates that DFs play an important role in enamel matrix formation and mineralization [26]. In this study,* WNT2* and* LEF1* were upregulated in DFs suggesting that DFs are involved in the complex interplay of signaling factors that regulate tooth initiation and morphogenesis [27, 28].* Runx2* is a key regulator of osteoblast marker genes and promotes the differentiation of mesenchymal stem cells into osteoblasts. The literature indicates that* Runx2* functions in the dental mesenchyme and mediates transduction signals from the dental epithelium to the mesenchyme during tooth development [29]. It also influences the molecular events that regulate tooth eruption—the most important physiologic role is likely being at the eruptive site [30]. Given the adaptive role of DFs, the presence of these genes suggests a central role of DFs in tooth formation.

Genes encoding protein modification- and signal transduction-related proteins tend to be expressed at higher levels in DFs than in the gingiva. The metalloprotease* ADAM 12* has been implicated in biological processes including fertilization and neurogenesis in DFs [11]. MMP-13 may be a major collagenolytic enzyme that degrades the extracellular matrix during tooth eruption. The upexpression of MMP-13 means DFs have important functions for the coordination of tooth eruption [31]. CXCL12 is a chemotactic factor for mesenchymal stem cells and mediates the suppressive effect of those cells on osteoclastogenesis. This factor can be expressed in DFs during tooth development including the epithelium surrounding the developing tooth bud [32].

To verify cDNA microarray results, six genes of different functions were selected for quantitative RT-PCR analyses. The expression levels of* KRT6A*,* CSTA*, and* CXCL10* were upregulated in the gingiva;* AMBN*,* ADAM12*, and* CXCL12* were upregulated in DFs. These results were consistent with the microarray results. To better understand the roles of the differentially expressed genes, IHC analysis was performed to identify their functions at tissue level.* CXCL10* and* CSTA* were strongly stained in all of the layers of the gingival tissue but were not stained in DFs. The genes that are highly expressed in the gingiva are stained in the epithelium because the prominent difference in structure between the gingiva and DFs is in the keratinized epithelium.* AMBN* and* CXCL12* were broadly stained in the outer area of DFs especially in the reduced enamel epithelium.

Several cell populations with stem cells properties have been isolated from different parts of dental tissue. Their participation in tissue repair and maintenance has been proposed [1]. Although it is difficult to characterize dental stem cells using surface protein markers, our results indicate the relative overexpression of important markers including CD13, CD34, CD73, and CD105 in DFs. These are ubiquitously expressed by all dental stem or precursor cells [6, 16]. With the exception of CD90, CD13, and CD34 which were frequently cited as dental-derived stem cell markers in previous studies, we selected CD106* (VCAM1)* and CD166* (ALCAM)*, which are expressed more strongly in dental follicles. Other dental-derived stem marker genes including CD29, CD90, and CD73 were expressed at higher levels indicating self-renewing and differentiation capacities in DFs [33].

Interestingly, the gingiva expressed high levels of iPS-associated markers* (OCT4*,* cMYC*, SOX2, and* KLK4)* versus DFs [34]. These proteins are transcription factors that are essential for maintaining the self-renewal capacity or pluripotency [35]. The iPS cells offer an advantage over traditional MSCs because they display an unlimited growth capacity that can serve as an inexhaustible source of stem cells [36]. A similar comparable report analyzed that dental tissue derived mesenchymal-like stem cells can be reprogrammed into iPSCs more efficiently, when compared to other mature somatic cells from human body such as adult MSCs and adult dermal fibroblasts [37].

The accessibility of dental tissue, including MSCs, might still be limited because these cells can only be isolated under specific circumstances, such as during the extraction of teeth. However, the gingiva is one of the most convenient tissues to collect by biopsy, with less scar formation and less postsurgical donor discomfort. In addition, gingival tissues are routinely resected during dental procedures in children, such as surgical extraction of impacted teeth and surgical opening for teeth with delayed eruption, and these tissues are generally treated as biomedical waste. In the laboratory, it is also feasible to isolate stem cells from gingival tissue based on their highly proliferative nature. Thus, the gingiva can be an important alternative source of stem cells in regenerative dentistry. If stem cells isolated from gingival tissue can be utilized similar to the storage of umbilical cord blood, the dynamic features of these cells reveal much potential for their use. Although this study is limited to monitoring expression patterns without a clinical link, comparative gene expression analysis of different tissues might provide genetic information concerning functions, such as tissue repair and tooth development. Further investigations are needed to evaluate the neurogenesis capacity, mineralization potential, and cell proliferation capacity of stem cells from gingiva and dental follicles based on of this study.

## 5. Conclusion

For the first time, this study profiles differential gene expression between the gingiva and DFs. cDNA microarray was performed to characterize and compare the molecular fingerprints of stemness. The DFs have been considered a multipotent tissue based on their ability to generate cementum, bone, and PDL. While the gingiva was not noticed for pluripotent stemness before, this study demonstrated transcription factors of iPS cells were expressed at higher levels in the gingiva and most dental-derived stem cell markers were strongly upregulated in the DFs. Given the minimal postsurgical discomfort and simple accessibility of gingival tissue, the gingiva is a good candidate stem cell source in regenerative dentistry.

## Figures and Tables

**Table 1:** Specific primer used for quantitative RT-PCR analysis.

Gene symbol	Functions	Assay ID	Product size (bp)
KRT6A	Ectoderm development, positive regulation of cell proliferation, cell differentiation	Hs01699178_g1	83
CXCL10	Positive regulation of leukocyte, chemotaxis	Hs01124251_g1	135
CSTA	Keratinocyte differentiation, negative regulation of peptidase activity	Hs00193257_m1	114
AMBN	Cell proliferation, bone mineralization, odontogenesis of dentin-containing tooth	Hs00212970_m1	61
ADAM12	Cell-cell and cell-matrix interactions, including fertilization, muscle development, neurogenesis	Hs01106101_m1	54
CXCL12	Immune response, positive regulation of monocyte chemotaxis	Hs03676656_mH	88
C-MYC	Regulation of transcription, DNA-dependent	Hs00153408_m1	107
KLF4	Mesodermal cell fate determination, negative regulation of cell proliferation, regulation of transcription	Hs00358836_m1	110
SOX2	Negative regulation of transcription from RNA polymerase II promoter, osteoblast differentiation	Hs01053049_s1	91
CD106	Response to hypoxia, acute inflammatory response, chronic inflammatory response	Hs01003372_m1	62
CD166	Cell adhesion, signal transduction, motor axon guidance	Hs00977641_m1	103
18S rRNA		Hs03003631_g1	69

**Table 2:** Representative genes differentially expressed with higher expression levels in the gingiva than in dental follicles (absolute fold change > 4.0).

Functional category	Gene symbol	Biological process	Accession number	Absolute fold change	Standard deviation
Metabolism and catabolism	LIPK	Lipid catabolic process	NM_001080518	90.99	11.87
	FMO2	Organic acid metabolic process	NM_001460	34.26	7.05
	ARG1	Arginine catabolic process	NM_000045	18.91	5.06
	LIPN	Lipid catabolic process	NM_001080518	13.27	4.19

Protein modification and maintenance	KLK7	Proteolysis	NM_139277	30.47	6.52
	KLK10	Proteolysis	NM_002776	28.97	6.34
	KLK6	Protein autoprocessing	NM_002774	25.58	6.10
	TGM1	Protein modification process	NM_000359	22.21	5.48
	OCLN	Protein complex assembly	NM_002538	12.48	4.48

Structural process	SPRR2A	Keratinization	NM_005988	207.84	18.61
	KRT1	Keratinization	NM_006121	146.08	15.41
	CNFN	Keratinization	NM_032488	74.92	10.64
	CSTA	Keratinocyte differentiation	NM_005213	69.63	10.22
	KRT4	Cytoskeleton organization	NM_002272	39.48	7.50
	KRT3	Cytoskeleton organization	NM_057088	36.71	7.23
	FLG	Keratinocyte differentiation	NM_002016	24.31	5.75
	DSP	Keratinocyte differentiation	NM_004415	17.15	5.22

Transport activity	CLCA4	Ion transport	NM_012128	48.96	8.48
	AQP3	Water transport	NM_004925	27.74	6.41
	SLC5A1	Transmembrane transport	NM_000343	19.52	5.09
	GLTP	Glycolipid transport	NM_016433	7.56	3.04

Developmental process	KRT10	Epidermis development	NM_000421	152.93	15.74
	SCEL	Epidermis development	NM_144777	134.38	14.68
	KRT6B	Ectoderm development	NM_005555	90.30	12.11
	KRT6A	Ectoderm development	NM_005554	57.61	9.64
	SPINK5	Epidermal cell differentiation	NM_001127698	55.60	9.34
	EHF	Epithelial cell differentiation	NM_012153	14.27	5.50
	SOX2	Embryonic development	NM_003106	8.67	3.34
	TUFT1	Odontogenesis	NM_020127	7.87	3.19

Physiologic process	RHCG	Regulation of pH	NM_016321	51.23	8.68
	ABCA12	Cellular homeostasis	NM_173076	39.33	7.55
	EREG	Angiogenesis	NM_001432	13.04	4.29
	NMU	Gastric acid secretion	NM_006681	12.72	4.05
	SCD	Oxidation reduction	NM_005063	4.35	2.25

Nucleic acid synthesis and modification	MACC1	Regulation of cell division	NM_182762	20.30	5.38
	ESRP1	mRNA processing	NM_017697	17.02	5.85
	HIST1H1B	Nucleosome assembly	NM_005322	6.85	2.91

Signal transduction and regulation	IL1F9	Cell-cell signaling	NM_019618	26.31	6.03
	ARAP2	Signal transduction	NM_015230	9.88	3.89
	DAPP1	Signal transduction	NM_014395	8.90	3.32

Apoptosis	MAL	Induction of apoptosis	NM_002371	49.41	8.48
	ALOX12	Antiapoptosis	NM_000697	31.70	6.69
	FAM3B	Apoptosis	NM_058186	27.28	6.16
	BNIPL	Apoptosis	NM_001159642	18.88	5.01

Cell adhesion	CLDN17	Cell-cell adhesion	NM_012131	91.67	11.90
	CRNN	Cell-cell adhesion	NM_016190	71.09	10.39
	DSC3	Homophilic cell adhesion	NM_024423	27.40	6.38
	CDSN	Cell adhesion	NM_001264	26.60	5.80
	DSG3	Cell adhesion	NM_001944	23.82	7.07

Cell cycle and transcriptional regulation	GRHL1	Regulation of transcription	NM_198182	31.32	6.62
	IRF6	Cell cycle arrest	NM_006147	13.05	4.87
	CASZ1	Regulation of transcription	NM_001079843	4.29	2.27
	E2F8	Regulation of transcription	NM_024680	4.20	2.21

Immune and inflammatory process	SERPINB4	Immune response	NM_002974	73.33	10.65
	IL1F6	Inflammatory response	NM_014440	43.13	7.87
	IL1RN	Inflammatory response	NM_173842	26.09	6.48
	IL1A	Inflammatory response	NM_000575	23.93	5.74
	CD1A	Immune response	NM_001763	4.16	2.19

Cytokine and chemokine activity	CXCL17	Chemotaxis	NM_198477	11.34	3.83
	CCL21	Chemotaxis	NM_002989	6.25	2.78
	ANLN	Cytokinesis	NM_018685	5.84	2.63
	CXCL10	Chemotaxis	NM_001565	4.29	2.37

**Table 3:** Representative genes differentially expressed with higher expression levels in dental follicles than in the gingiva (absolute fold change > 4.0).

Functional category	Gene symbol	Biological process	Accession number	Absolute fold change	Standard deviation
Metabolism and catabolism	ALDH1L2	Carbon metabolic process	NM_001034173	19.63	5.13
	MOXD1	Histidine catabolic process	NM_015529	17.92	4.91
	ELOVL2	Fatty acid metabolic process	NM_017770	12.62	4.07
	FBXL7	Protein catabolic process	NM_012304	8.58	3.27

Protein modification and maintenance	ADAM12	Metalloendopeptidase activity	NM_003474	37.09	7.25
	MMP16	Metalloendopeptidase activity	NM_005941	24.32	5.82
	MMP2	Metalloendopeptidase activity	NM_004530	19.64	5.17
	MMP8	Metalloendopeptidase activity	NM_002424	11.86	3.89
	MMP13	Metalloendopeptidase activity	NM_002427	7.60	3.16
	ADAM22	Proteolysis	NM_021723	5.97	2.75

Structural process	COL11A1	Extracellular matrix organization	NM_001854	29.15	6.38
	MAP1B	Microtubule bundle formation	NM_005909	10.30	3.61
	FBN2	Anatomical structure morphogenesis	NM_001999	9.02	3.40
	LUM	Collagen fibril organization	NM_002345	8.68	3.32

Transport activity	KCNT2	Ion transport	NM_198503	11.30	3.80
	ABCC9	Potassium ion transport	NM_005691	11.18	3.77
	RHOBTB3	Retrograde transport	NM_014899	10.62	3.72
	SLC4A4	Sodium ion transport	NM_001098484	10.12	3.68
	HEPH	Copper ion transport	NM_138737	8.34	3.28

Developmental process	AMBN	Odontogenesis	NM_016519	117.54	16.99
	CDH11	Ossification	NM_001797	38.12	7.40
	ALPL	Biomineral tissue development	NM_000478	33.21	6.83
	ASPN	Bone mineralization	NM_017680	33.05	6.85
	FGF7	Embryonic development	NM_002009	29.53	6.44
	COL1A2	Skeletal system development	NM_000089	14.50	4.41
	RUNX2	Ossification	NM_001024630	13.85	4.23
	PDGFRB	Embryonic development	NM_002609	11.85	3.93
	WNT2	Mesenchymal cell proliferation	NM_003391	10.28	3.73
	BMP5	Ossification	NM_021073	7.13	3.28
	LEF1	Wnt receptor signaling pathway	NM_016269	5.83	2.66
	PAX3	Organ morphogenesis	NM_181457	4.70	2.38
	MSX1	Organ morphogenesis	NM_002448	4.23	2.24

Physiologic process	VAT1L	Oxidation reduction	NM_020927	12.30	3.98
	TFPI	Blood coagulation	NM_006287	9.49	3.49
	TPM1	Muscle contraction	NM_000366	8.78	3.30
	SOBP	Sensory perception	NM_018013	8.27	3.21

Nucleic acid synthesis and modification	EYA4	DNA repair	NM_004100	24.90	5.86
	NAP1L3	Nucleosome assembly	NM_004538	16.47	4.68
	SNRPN	RNA splicing	BC043194	5.05	1.58

Signal transduction and regulation	PDE7B	Signal transduction	NM_018945	22.99	5.59
	CHN1	Signal transduction	NM_018945	22.98	5.60
	LIFR	Cytokine-mediated signaling pathway	NM_002310	8.78	3.31
	FSTL1	BMP signaling pathway	NM_007085	8.75	3.31

Apoptosis	SEMA3A	Apoptosis	NM_006080	51.87	8.72
	PEG10	Apoptosis	NM_015068	21.89	5.43
	SULF1	Apoptosis	NM_001128205	11.18	3.77
	NELL1	Induction of apoptosis	NM_006157	8.67	3.27

Cell adhesion	OMD	Cell adhesion	NM_005014	40.83	7.69
	VCAN	Cell adhesion	NM_004385	35.76	7.25
	SPON1	Cell adhesion	NM_006108	32.63	6.78

Cell cycle and transcriptional regulation	MYEF2	Transcription	NM_016132	6.71	2.88
	SYCP2	Cell cycle	NM_014258	5.41	2.53
	APBB2	Cell cycle arrest	NM_004307	5.25	2.49

Immune and inflammatory process	TPST1	Inflammatory response	NM_003596	9.00	3.34
	PXDN	Immune response	NM_012293	8.89	3.40
	IFI44L	Immune response	NM_006820	6.01	2.79
	PECAM1	Phagocytosis	NM_000442	4.26	2.25
	COLEC12	Phagocytosis, recognition	NM_130386	4.23	2.22

Cytokine and chemokine activity	CXCL12	Chemotaxis	NM_000609	11.04	3.79
	SLIT3	Chemotaxis	NM_003062	8.94	3.34
	CMTM3	Chemotaxis	NM_144601	5.24	2.52
	STX2	Cytokinesis	NM_194356	4.39	2.27
	CCR1	Chemotaxis	NM_001295	4.31	2.36
